# PeriOperative Quality Initiative (POQI) international consensus statement on perioperative arterial pressure management

**DOI:** 10.1016/j.bja.2024.04.046

**Published:** 2024-06-04

**Authors:** Bernd Saugel, Nick Fletcher, Tong J. Gan, Michael P.W. Grocott, Paul S. Myles, Daniel I. Sessler, PeriOperative Quality Initiative XI (POQI XI) Workgroup Members

**Affiliations:** 1 Department of Anesthesiology, Center of Anesthesiology and Intensive Care Medicine, University Medical Center Hamburg-Eppendorf, Hamburg, Germany; 2 Outcomes Research Consortium, Cleveland, OH, USA; 3 Institute of Anesthesia and Critical Care, Cleveland Clinic London, London, UK; 4 Division of Anesthesiology and Perioperative Medicine, Critical Care and Pain Medicine, The University of Texas MD Anderson Cancer Center, Houston, TX, USA; 5 Perioperative and Critical Care Theme, NIHR Southampton Biomedical Research Centre, University Hospital Southampton NHS Foundation Trust/University of Southampton, Southampton, UK; 6 Department of Anaesthesiology and Perioperative Medicine, Alfred Hospital and Monash University, Melbourne, VIC, Australia; 7 Outcomes Research Consortium, Department of Anesthesiology, Cleveland Clinic, Cleveland, OH, USA

**Keywords:** anaesthesia, arterial pressure, cardiovascular dynamics, complications, guidelines, haemodynamic monitoring, hypotension, postoperative outcome

## Abstract

Arterial pressure monitoring and management are mainstays of haemodynamic therapy in patients having surgery. This article presents updated consensus statements and recommendations on perioperative arterial pressure management developed during the 11th POQI PeriOperative Quality Initiative (POQI) consensus conference held in London, UK, on June 4–6, 2023, which included a diverse group of international experts. Based on a modified Delphi approach, we recommend keeping intraoperative mean arterial pressure ≥60 mm Hg in at-risk patients. We further recommend increasing mean arterial pressure targets when venous or compartment pressures are elevated and treating hypotension based on presumed underlying causes. When intraoperative hypertension is treated, we recommend doing so carefully to avoid hypotension. Clinicians should consider continuous intraoperative arterial pressure monitoring as it can help reduce the severity and duration of hypotension compared to intermittent arterial pressure monitoring. Postoperative hypotension is often unrecognised and might be more important than intraoperative hypotension because it is often prolonged and untreated. Future research should focus on identifying patient-specific and organ-specific hypotension harm thresholds and optimal treatment strategies for intraoperative hypotension including choice of vasopressors. Research is also needed to guide monitoring and management strategies for recognising, preventing, and treating postoperative hypotension.

**Editor's key points**

An international panel of experts updated previous consensus guidelines for managing intraoperative arterial pressure using a modified Delphi approach, and recommend the following:

• As intraoperative mean arterial pressures <60–70 mm Hg or systolic arterial pressures <90–100 mm Hg are associated with acute kidney injury, myocardial injury, myocardial infarction, and death, intraoperative mean arterial pressure should be maintained ≥60 mm Hg in at-risk patients.
• Mean arterial pressure targets should be increased when venous or compartment pressures are elevated.
• Hypotension treatment should be based on underlying causes including vasodilation, hypovolaemia, bradycardia, and low cardiac output.
• If intraoperative hypertension is treated, hypotension should be avoided.
• Continuous intraoperative arterial pressure monitoring helps reduce the severity and duration of hypotension compared to intermittent arterial pressure monitoring.
• Postoperative hypotension is often unrecognised and might be more important than intraoperative hypotension because it is often prolonged.
• Further research should investigate patient-specific and organ-specific harm thresholds for hypotension and optimal therapies.

Systemic arterial pressure results from the interaction between cardiac output and systemic vascular resistance; it is characterised by systolic, mean, and diastolic components. Low arterial pressure, or hypotension, is common during surgery.^1^ Although harm thresholds remain unclear, hypotension at some level causes organ injury, complications, and death.^2^ Arterial pressure monitoring and management thus are mainstays of haemodynamic therapy in patients having surgery.^3^

In 2019, four articles^4^, ^5^, ^6^, ^7^ summarised consensus statements and practice recommendations of the third PeriOperative Quality Initiative (POQI) consensus conference on perioperative arterial pressure management held in 2017. Here, we present updated consensus recommendations on perioperative arterial pressure management based on recent information.

## Methods

POQI is an international multidisciplinary non-profit organisation that organises consensus conferences on clinical key topics related to perioperative medicine.^8^ Manuscripts and key figures from previous meetings are available on the POQI website (www.thepoqi.org). POQI conferences assemble a collaborative group of diverse international experts to develop consensus-based recommendations in perioperative medicine. On June 4–6, 2023, the 11th POQI meeting took place in London, UK, to update recommendations in three key areas in perioperative medicine: perioperative arterial pressure management, goal-directed haemodynamic therapy, and fluid therapy. An international panel of experts in perioperative medicine from Europe, North America, and Australia reviewed previously published recommendations and provided updated consensus recommendations based on new literature published in each field. Consensus was achieved using a modified Delphi method, designed to use the collective expertise of a diverse group of experts to answer clinically important questions using published methods.^9^

In the pre-meeting phase, we reviewed previous consensus statements and recommendations and searched and reviewed relevant literature published since the previous consensus statements.^4^, ^5^, ^6^, ^7^ Specifically, we searched PubMed for articles on intraoperative and postoperative arterial pressure published between January 2019 and June 2023 using the following search terms: ((hypotension[tiab] OR hypotensive[tiab]) AND (intraoperative[tiab] OR perioperative[tiab]) AND (blood pressure[tiab])). Additionally, we searched the reference lists of articles. We restricted the search to trials, studies, reviews, and meta-analyses published in English. The search resulted in 369 articles, including 42 interventional trials and 10 meta-analyses. We additionally searched ClinicalTrials.gov and the Cochrane database and asked experts to nominate additional relevant articles for consideration.

During the 11th POQI meeting, the arterial pressure management group (the authors of this article) formulated the main statements and recommendations that were then iteratively reviewed and revised between alternating small group (arterial pressure management group only) and plenary (all panellists of the 11th POQI meeting) sessions with voting to approve (or not) in the final plenary session. The votes on all our final statements and recommendations were unanimous. The Grading of Recommendations, Assessment, Development, and Evaluations (GRADE) framework was used to categorise the quality of evidence and the strength of recommendations.^10^ Areas of inadequate knowledge and unanswered questions were also identified and highlighted. After the 11th POQI meeting, on July 6, 2023, we additionally presented our recommendations at Evidence Based Perioperative Medicine (EBPOM) 2023 World Congress in London, UK, and invited attendees to participate in an anonymous voting indicating their agreement or disagreement. The results of this voting are presented in Table 1.

**Table 1:** Anonymous voting of attendees of the Evidence Based Perioperative Medicine (EBPOM) 2023 World Congress on the recommendations.

Recommendation	Number of votes (n)	Agreement (%)
***Consensus recommendation 1:*** We recommend keeping intraoperative mean arterial pressure ≥60 mm Hg in at-risk patients.	100	97
***Consensus recommendation 2:*** We recommend increasing mean arterial pressure targets when venous or compartment pressures are elevated.	99	94
***Consensus recommendation 3:*** We recommend that treatment of hypotension be based on presumed underlying causes including vasodilation, hypovolaemia, bradycardia, and low cardiac output.	103	100
***Consensus recommendation 4:*** If intraoperative hypertension is treated, we recommend caution to avoid hypotension.	102	100

After the meeting, we prepared and revised the manuscript reporting this process. This article summarises the consensus statements and recommendations of the perioperative arterial pressure management group and is structured considering key aspects of the Appraisal of Guidelines, Research and Evaluation (AGREE) II statement.^11^

## Consensus statements and recommendations

***Consensus statement 1:*** Intraoperative mean arterial pressures <60–70 mm Hg or systolic arterial pressures <90–100 mm Hg are associated with acute kidney injury, myocardial injury, myocardial infarction, and death. Injury is a function of hypotension severity and duration (high-quality evidence).

***Consensus recommendation 1:*** We recommend keeping intraoperative mean arterial pressure ≥60 mm Hg in at-risk patients (strong recommendation, moderate-quality evidence).

Intraoperative hypotension remains vaguely defined. Consequently, definitions for intraoperative hypotension vary widely.^12^ The most commonly used definitions are a systolic arterial pressure <90 mm Hg or a mean arterial pressure <60 mm Hg.^12^ A 20% reduction from baseline systolic or mean arterial pressure is also frequently used,^12^ although it remains unclear how to best define baseline arterial pressure. Arterial pressure measured just before induction of anaesthesia does not reflect normal baseline arterial pressures.^13^ Even clinic arterial pressures can be inaccurate. The best assessment of baseline arterial pressure is preoperative ambulatory arterial pressure monitoring,^4^ although it is rarely available.

Recent observational research confirms previous studies^14^, ^15^, ^16^, ^17^, ^18^, ^19^ in showing that, on a population basis, intraoperative mean arterial pressures <60–70 mm Hg or systolic arterial pressures <90–100 mm Hg are associated with acute kidney injury,^20^, ^21^, ^22^, ^23^, ^24^ myocardial injury,^21^ myocardial infarction,^22^^,^^25^^,^^26^ and death.^22^ Injury is a function of hypotension severity and duration. Harm from hypotension apparently largely accrues during brief periods of profoundly low arterial pressures rather than from prolonged exposure to moderately low arterial pressures.^27^ Absolute maximum decrease in arterial pressure and area under arterial pressure thresholds, which considers both severity and duration of hypotension, appear most strongly associated with organ injury.^28^ Associations with patient harm are similar for systolic and mean arterial pressures.^21^

Baseline arterial pressure varies considerably among individual patients presenting for surgery.^13^ Intraoperative hypotension harm thresholds presumably also differ among individuals. Baseline patient risk factors are far more strongly associated with postoperative organ injury than intraoperative hypotension.^21^^,^^23^ But hypotension, in contrast to most baseline risk factors, is potentially modifiable and therefore of considerable interest.

The associations between intraoperative hypotension and acute kidney and myocardial injury are clear. However, it remains largely unknown whether the observed associations are causal, and thus amenable to intervention. Only a few randomised trials have reported the effect of targeted arterial pressure management on postoperative outcomes.^29^

A single-centre trial of 458 major noncardiac surgery patients with high baseline cardiovascular risk tested the hypothesis that keeping intraoperative mean arterial pressure ≥75 mm Hg compared to ≥60 mm Hg reduces the incidence of a composite primary outcome of acute myocardial injury within the first 3 postoperative days and 30-day acute kidney injury or major adverse cardiovascular events.^30^ Patients assigned to the ≥75 mm Hg mean arterial pressure target spent substantially less time with hypotension than patients assigned to the ≥60 mm Hg mean arterial pressure target (e.g. median cumulative time with a mean arterial pressure <65 mm Hg 9 *vs* 23 min).^30^ However, the incidence of the primary composite outcome was similar in each group: 48% in patients assigned to the ≥75 mm Hg mean arterial pressure target and 52% in patients assigned to the ≥60 mm Hg mean arterial pressure target (risk difference –4.2%, 95% confidence interval [CI] –13% to 5%; *P*=0.42).

The large international POISE-3 trial tested the effects of a hypotension-avoidance *vs* a hypertension-avoidance strategy on major postoperative vascular complications in 7490 patients with hypertension having noncardiac surgery.^31^ The intraoperative target mean arterial pressures were ≥80 mm Hg in the hypotension-avoidance group and ≥60 mm Hg in the hypertension-avoidance group. The median time with intraoperative mean arterial pressure of 60–79 mm Hg was 25 min in patients in the hypotension-avoidance group and 56 min in patients in the hypertension-avoidance group.^31^ However, profound hypotension was apparently rare in both groups. Absolute maximum arterial pressure declines and areas under arterial pressure thresholds were not reported. The composite primary outcome (vascular death and nonfatal myocardial injury after noncardiac surgery, stroke, and cardiac arrest within the first 30 days after surgery) occurred in 14% of patients assigned to the hypotension-avoidance group and in 14% of patients assigned to the hypertension-avoidance group (hazard ratio 0.99, 95% CI 0.88 to 1.12; *P*=0.92).^31^

These two trials^30^^,^^31^ suggest that targeting mean arterial pressures >60 mm Hg does not prevent organ injury. The ongoing GUARDIAN trial (NCT04884802), with a target enrolment of 6250 high-risk patients, is testing the hypothesis that keeping intraoperative mean arterial pressure ≥85 mm Hg *vs* routine arterial pressure management improves a composite of serious perfusion-related complications.

Inter-individual variability in preoperative baseline arterial pressure^13^ might justify individualising intraoperative arterial pressure targets. The INPRESS trial^32^ tested the hypothesis that individualised arterial pressure management improves postoperative outcomes compared to routine arterial pressure management in 298 patients who had major, predominantly abdominal, noncardiac surgery.^32^ The composite primary outcome of systemic inflammatory response syndrome and dysfunction of at least one major organ system within 7 days after surgery occurred in 38% of patients assigned to individualised management and in 52% of patients assigned to routine management (relative risk 0.73; 95% CI 0.56 to 0.94; *P*=0.02).^32^ In patients assigned to individualised management, norepinephrine was continuously administered during surgery to keep systolic arterial pressures within 10% of the preoperative resting value.^32^ In contrast, in patients in the routine management group, ephedrine boluses were only given for systolic arterial pressure <80 mm Hg or >40% below preoperative values.^32^

The ongoing IMPROVE-multi trial^33^ (NCT05416944), with a target enrolment of 1272 patients, is testing the hypothesis that personalised perioperative arterial pressure management, based on preoperative night-time mean arterial pressure assessed using automated arterial pressure monitoring, reduces the incidence of a composite outcome of acute kidney injury, acute myocardial injury, nonfatal cardiac arrest, and death within 7 days after surgery compared to routine arterial pressure management in high-risk patients having major abdominal surgery.^33^

In summary, there are clear associations between intraoperative hypotension and renal and myocardial injury. However, trial data remain sparse and suffer methodologic limitations. The extent to which the associations are causal thus remains largely unknown; consequently, the acceptable lower limit of intraoperative arterial pressure remains uncertain. Nonetheless, available information suggests that keeping intraoperative mean arterial pressure ≥60 mm Hg is advisable.

***Consensus recommendation 2:*** We recommend increasing mean arterial pressure targets when venous or compartment pressures are elevated (strong recommendation, moderate-quality evidence).

Mean arterial pressure, the mean pressure over the cardiac cycle, is the inflow pressure for most organs and therefore a main determinant of organ perfusion pressure.^3^, ^4^ However, organ perfusion pressure is also influenced by venous outflow pressure and extravascular pressure in the relevant tissue. Organ perfusion pressure can be substantially reduced by increased extravascular compartment pressures in the abdomen, thorax, limbs, and cranium. Venous pressure can be elevated by right ventricular failure, systemic arteriovenous shunts, and venous obstruction.

The kidneys appear particularly sensitive to perioperative decreases in perfusion pressure.^34^, ^35^, ^36^, ^37^ Elevated intraabdominal pressure promotes splanchnic venous congestion and impairs renal function.^38^ During surgery, venous pressure is often unknown, but operative factors including steep Trendelenburg (head-down) positioning and peritoneal or thoracic gas insufflation increase organ outflow pressure, possibly resulting in inadequate organ perfusion. We, therefore, recommend compensating for estimated venous outflow pressure and extravascular pressure on the relevant tissue when defining individual intraoperative mean arterial pressure targets.

However, there are no trials investigating how to best correct for increased venous outflow pressure or extravascular pressure. In clinical practice, venous and compartment pressures are often unknown. Central venous pressure can be measured in surgical patients with a central venous catheter, but compartment pressures (such as intraabdominal and intracranial pressures) are generally unknown. If compartment pressure is available or can be estimated, pathophysiological rationale suggests increasing the mean arterial pressure target by roughly the compartment pressure. For example, if clinicians aim for an organ perfusion pressure of 65 mm Hg and compartment pressure is 15 mm Hg, the inflow pressure (i.e. mean arterial pressure) might be maintained >80 mm Hg.

***Consensus recommendation 3:*** We recommend that treatment of hypotension be based on presumed underlying causes including vasodilation, hypovolaemia, bradycardia, and low cardiac output (strong recommendation, high-quality evidence).

Hypotension can result from several underlying physiological processes acting alone or in combination.^39^ Vasodilation, hypovolaemia, bradycardia, and low cardiac output are all modifiable causes of hypotension.^39^ Vasodilation can be reversed by vasopressors such as phenylephrine or norepinephrine. Hypovolaemia can be treated with intravascular fluid administration using a variety of different types of fluids including crystalloid and colloid solutions or blood. Bradycardia is typically pharmacologically managed with anticholinergic agents such as atropine or glycopyrronium; when not responsive to these agents, epinephrine or isoprenaline might be necessary. A pacemaker can be used to manage profound bradycardia. Finally, low cardiac output due to acute or chronic myocardial dysfunction can be treated with positive inotropic agents such as dobutamine or epinephrine.

***Consensus statement 2:*** In general surgery, there is no association between intraoperative systolic arterial pressures between 120 and 200 mm Hg and acute kidney injury and myocardial injury (low-quality evidence).

***Consensus recommendation 4:*** If intraoperative hypertension is treated, we recommend caution to avoid hypotension (strong recommendation, moderate-quality evidence).

A single-centre observational analysis reported no relationship between intraoperative systolic arterial pressures of 120–200 mm Hg and myocardial injury.^40^ In contrast, many analyses report associations between hypotension and myocardial injury,^18^^,^^41^ myocardial infarction,^42^ acute kidney injury,^18^ and death,^16^ although trial evidence remains equivocal.^30^, ^31^, ^32^

Some degree of hypertension should prompt treatment. However, given that reasonable levels of hypertension appear relatively benign, available data suggest that hypertension should be treated incrementally, if at all, with the goal of avoiding hypotension, which undoubtedly causes organ injury at some level.

***Consensus statement 3:*** Continuous intraoperative arterial pressure monitoring helps clinicians reduce the severity and duration of hypotension compared to intermittent arterial pressure monitoring (high-quality evidence).

Arterial catheters are used to monitor haemodynamic fluctuations during complex operations and in high-risk patients with significant comorbidities.^43^ However, oscillometric monitoring at 2–5-min intervals is most often used in healthy patients having low- or moderate-risk procedures.^44^, ^45^, ^46^ Innovative methods now allow noninvasive continuous arterial pressure monitoring.^44^^,^^47^, ^48^, ^49^

There is convincing evidence that continuous compared to intermittent arterial pressure monitoring identifies more intraoperative hypotension, thereby helping clinicians reduce the duration and severity of hypotension.^50^ During induction of general anaesthesia, continuous intraarterial arterial pressure monitoring, compared to intermittent oscillometric (with blinded intraarterial) arterial pressure monitoring, helped clinicians reduce the amount of hypotension (area under a mean arterial pressure of 65 mm Hg) by a factor of ∼3 in a single-centre trial.^51^ Clinicians should thus consider inserting an arterial catheter before, rather than after, induction of anaesthesia in patients for whom continuous intraarterial arterial pressure monitoring is planned. In a similar single-centre trial, using continuous finger-cuff *vs* intermittent oscillometric (with blinded continuous finger-cuff) arterial pressure monitoring also resulted in substantially less hypotension during induction of anaesthesia.^52^

During surgery, continuous arterial pressure monitoring with an arterial catheter detected nearly twice as much hypotension as oscillometric arterial pressure monitoring in a randomised trial in 306 patients having noncardiac surgery.^53^ Continuous finger-cuff monitoring reduced intraoperative hypotension (quantified as the time-weighted average mean arterial pressure <65 mm Hg) compared to intermittent oscillometric monitoring by a factor of 2 in a trial of 316 noncardiac surgical patients,^54^ and by a factor of 10 in another trial of 242 patients.^52^ In summary, there is strong evidence that continuous arterial pressure monitoring allows clinicians to intervene early and effectively, thus substantially reducing intraoperative hypotension.

***Consensus statement 4:*** Postoperative hypotension is often unrecognised and might be more important than intraoperative hypotension because it is often prolonged (moderate-quality evidence). However, postoperative hypotensive harm thresholds remain unclear.

Hypotension is common in postoperative patients because of antihypertensive medications, inadequate intravenous fluid administration, adverse effects of some anaesthetic drugs, intraoperative and ongoing blood loss, the inflammatory response to surgery, arrhythmias, and impaired myocardial function. Most hospitals therefore establish guidance on when to alert medical staff, for example, when systolic arterial pressure is <90 mm Hg.^55^ Postoperative hypotension often goes undetected, and lasts longer than intraoperative hypotension.^56^

Large observational studies have identified associations between postoperative hypotension and organ injury, especially acute kidney injury, cardiovascular events, readmission, and mortality.^57^, ^58^, ^59^, ^60^, ^61^ Harm thresholds appear to be an absolute systolic arterial pressure of 90–100 mm Hg or a mean arterial pressure of 60–75 mm Hg. Longer cumulative duration of postoperative hypotension is associated with higher risk.^58^

A challenge is that it has proven difficult to modify postoperative arterial pressures. For example, a delayed restart of chronic antihypertensive medications has no appreciable effect on ward arterial pressures.^31^ Trials show that avoiding beta blockers^62^ and clonidine^56^ reduces the risk of hypotension. Observational analyses suggest that avoiding angiotensin-converting enzyme inhibitors and angiotensin receptor blockers also reduces hypotension.^61^ However, in the SPACE trial,^63^ a six-centre trial of 262 patients aged ≥60 yr who had elective noncardiac surgery, discontinuing *vs* continuing angiotensin-converting enzyme inhibitors and angiotensin receptor blockers did not reduce myocardial injury or hypotension within 48 h of surgery. Beta blockers reduce the risk of myocardial injury but cause hypotension (and strokes).^62^ Avoiding clonidine does not reduce organ injury despite less severe hypotension.^56^ The effects of avoiding perioperative angiotensin-converting enzyme inhibitors and angiotensin receptor blockers need to be evaluated in further trials. It thus remains unclear whether postoperative hypotension is causally related to organ injury.

## Recommendations for research

**Fig 1:**
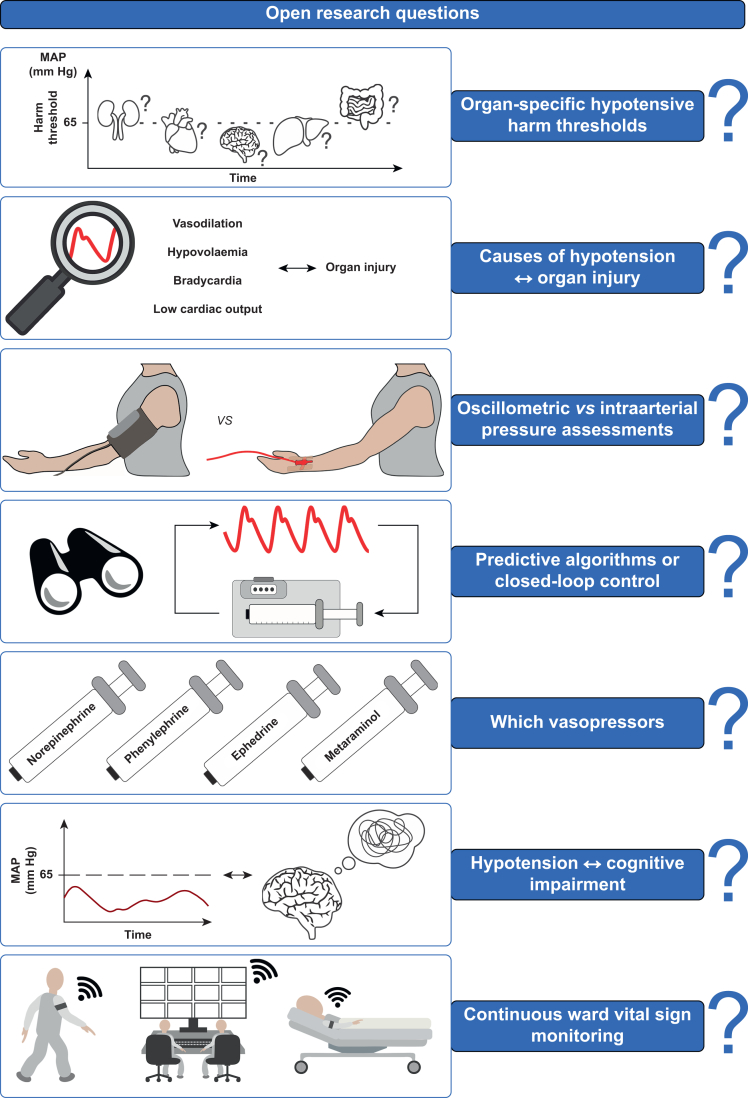
Open research questions in the field of perioperative arterial pressure management. MAP, mean arterial pressure.

It remains poorly understood what constitutes clinically important hypotension for individual patients, both during and after surgery. As a corollary, optimal strategies to avoid or treat intraoperative and postoperative hypotension remain to be investigated and rigorously tested. We consider the following questions important to be addressed in future research (Fig. 1).

### Are there organ-specific hypotensive harm thresholds for the kidney, heart, intestines, liver, and brain? Are hypotensive harm thresholds altered by perioperative factors? What arterial pressure threshold minimises risk to all organs?

For a given organ, the lower limit of blood flow autoregulation (i.e. the perfusion pressure below which organ blood flow becomes pressure dependent) defines clinically important hypotension.^64^ Despite the physiological importance of blood flow autoregulation, little is known about intraoperative lower limits of autoregulation of different organs, or how perioperative factors modify such thresholds. Although there is compelling evidence from observational research that intraoperative hypotension harm thresholds for acute kidney injury and myocardial injury are similar at mean arterial pressures of ∼65 mm Hg,^2^^,^^21^ harm thresholds for stroke, delirium, mesenteric ischaemia, or liver failure remain largely unknown.^2^ Harm thresholds presumably differ for various organs and vary among individuals based on overall health, age, specific medical conditions, and their treatments. Consequently, there is no universal arterial pressure threshold that ensures minimal risk to all organs. How general anaesthesia and surgical trauma with inflammation influence organ-specific hypotension harm thresholds is poorly investigated. It is thus currently unclear what the organ-specific hypotension harm thresholds are, and further research is needed.

### Do various causes of hypotension (vasodilation, hypovolaemia, bradycardia, or low cardiac output) affect the association between hypotension and organ injury?

Multiple factors contribute to intraoperative hypotension including anaesthetic drugs (which induce vasodilation, myocardial depression, or bradycardia), bleeding (resulting in intravascular hypovolaemia), mechanical ventilation (causing high intrathoracic pressure impairing venous return), and patient positioning. Consequently, there are various endotypes of intraoperative hypotension characterised by different underlying haemodynamic alterations. Identified endotypes include myocardial depression, bradycardia, vasodilation, and hypovolaemia.^39^ It remains unknown whether various causes of hypotension affect the association between hypotension and organ injury, although it seems likely that the degree of hypotension is more important than its cause. In contrast, treatment of hypotension should be directed to the presumed cause(s) because cause-specific treatments are more likely to be effective than generic treatments.

### Do hypotensive harm thresholds differ with oscillometric vs intraarterial arterial pressure measurements?

Direct intraarterial measurement from an arterial catheter is the clinical reference method for monitoring arterial pressure.^43^ Continuous intraarterial monitoring provides arterial pressure in real-time and helps clinicians detect and treat arterial pressure changes immediately. However, intraarterial monitoring is invasive, and very rarely causes serious complications such as ischaemia or major bleeding.^65^, ^66^, ^67^ Intraarterial monitoring is therefore indicated only in high-risk patients and those having major surgery. Arterial pressure is thus usually measured oscillometrically at 2–5-min intervals.

Oscillometric arterial pressure measurements are less accurate than generally assumed. For example, oscillometry substantially overestimates low arterial pressures and underestimates high arterial pressures, thus potentially missing clinically meaningful hypotension and hypertension.^68^ A consequence is that hypotensive harm thresholds for organ injury could differ with each arterial pressure measurement technique. For example, hypotensive harm thresholds might be higher with oscillometric than intraarterial measurements because oscillometry overestimates low arterial pressures.

### Do predictive algorithms or closed-loop control prevent organ injury?

Clinically apparent hypotension can be preceded by subtle changes in haemodynamic variables.^69^, ^70^, ^71^, ^72^ There have thus been various attempts to predict hypotension by analysing haemodynamic variables and identifying patterns of cardiovascular dynamics preceding hypotensive episodes. Machine learning-based algorithms that predict future changes in arterial pressure have received regulatory approval and are now commercially available.

For example, the Hypotension Prediction Index software (HPI-software; Edwards Lifesciences, Irvine, CA, USA) predicts hypotension by analysing invasively^73^ or noninvasively^74^ arterial pressure waveforms. Propensity-matched cohort^75^ and registry^76^ studies suggest that clinical implementation of this technology could help clinicians reduce intraoperative hypotension during noncardiac surgery. Trials investigating HPI-software monitoring show contradictory results: while a small trial suggested that HPI-software monitoring reduced hypotension,^77^ a larger trial did not.^78^ Robust randomised trials reporting relevant clinical outcomes are currently not available; consequently, the clinical benefit remains uncertain. In addition, there is ongoing debate about the adequacy of HPI-software validation^79^, ^80^, ^81^ and on whether HPI values simply reflect changes in mean arterial pressure.^82^^,^^83^

Automated closed-loop vasopressor administration has been shown to be feasible and effective in tightly maintaining arterial pressure at predefined target levels during surgery.^84^^,^^85^ In a small trial, closed-loop vasopressor administration reduced intraoperative hypotension compared to manual titration of norepinephrine infusion in patients having abdominal or orthopaedic surgery.^86^ Larger trials with relevant clinical outcomes are awaited.

### Which vasopressor best protects organs from hypotensive injury?

Vasopressors are usually effective and increase arterial pressure as intended. However, the physiologic goal of increasing arterial pressure is to increase the perfusion of sensitive organs, which is not solely determined by pressure.^87^ It is thus possible that pure vasopressors such as phenylephrine simultaneously increase arterial pressure and reduce organ perfusion. In contrast, combined α_1_-and β_1_-adrenergic agonists such as norepinephrine that maintain or improve cardiac output might improve organ perfusion. A further consideration is that ephedrine is subject to tachyphylaxis.^88^ Phenylephrine can induce reflex bradycardia and thus decrease cardiac output.^89^ Randomised trials are needed to establish whether perioperative norepinephrine provides superior organ protection and improved outcomes after major surgery. Feasibility trials have been completed^90^^,^^91^ and a large trial is underway (NCT04884802).

### Are intraoperative or postoperative hypotension associated with acute and long-term cognitive impairment?

A long-standing hypothesis is that intraoperative brain hypoperfusion promotes postoperative neurocognitive disorders such as postoperative delirium or delayed neurocognitive recovery. Retrospective studies of postoperative delirium suggest a link with intraoperative hypotension, although they likely suffered from incomplete adjustment for important confounders including age, frailty, and pre-existing cognitive impairment.^92^, ^93^, ^94^, ^95^

There are few trials of targeted intraoperative arterial pressure management and postoperative neurocognitive disorders.^96^^,^^97^ In a pilot trial of 101 patients ≥75 yr old having noncardiac surgery, personalised targeted *vs* untargeted intraoperative arterial pressure management did not reduce the incidence of postoperative delirium or cognitive dysfunction at 3 months.^96^ In contrast, a multicentre trial of 322 patients ≥65 yr old having noncardiac surgery reported a 50% relative reduction in the incidence of postoperative delirium within a week of surgery when targeting intraoperative mean arterial pressures of 95–100 mm Hg compared to 60–70 mm Hg.^97^

It thus remains unknown whether targeted intraoperative arterial pressure management reduces postoperative neurocognitive disorders.^94^^,^^95^^,^^98^, ^99^, ^100^ As preoperative cognitive status seems to be the most important determinant of postoperative neurocognitive disorders, robust trials on targeted arterial pressure management and postoperative neurocognitive disorders in older at-risk patients are needed.

### Does continuous ward vital sign monitoring allow clinicians to intervene in ways that improve patient outcomes?

When patients having major surgery reach the postanaesthesia care unit, families naturally assume that they have survived the most dangerous part of the perioperative experience. This assumption is wrong. Mortality in the 30 days after surgery is 140 times higher than intraoperative mortality.^101^^,^^102^ In fact, if the month after surgery were considered a disease, it would be the world's third leading cause of death.^103^

The most common causes of 30-day postoperative mortality are major bleeding^104^ and cardiopulmonary complications.^105^^,^^106^ Myocardial injury and infarction are strongly related to both intraoperative and postoperative hypotension. Respiratory complications are also common and are of special interest because nearly all are preventable.^107^

Ward hypotension, hypertension, and hypoxaemia are common, profound, and prolonged, and are nearly always missed by conventional vital sign monitoring at 4-h intervals.^108^, ^109^, ^110^ For example, the reported incidence of ward respiratory compromise is 0.3%–3.4% when defined by interventions such as naloxone administration,^111^ but is 21% when defined by prolonged oxygen desaturation^108^ and 41% when defined by bradypnea episodes.^112^ Nearly all these events are missed by routine 4-h nursing checks.^108^^,^^109^

Cardiopulmonary events do not occur in isolation. Tachycardia and hypoxaemia commonly coexist, and often culminate in hypotension which is strongly associated with myocardial injury and death.^14^^,^^16^ Vital signs usually deteriorate 6–12 h before cardiac and respiratory arrests occur,^113^, ^114^, ^115^ which is the basis for having hospital rapid-response teams.^116^ For example, continuous monitoring might speed the detection and treatment of sepsis.^117^ About 60% of critical events such as death and unplanned intensive care unit admission are preceded by clear abnormalities.^118^, ^119^, ^120^

The difficulty is that rapid response teams largely prevent further damage *after* patients experience critical events; patients would be better served if we could detect deterioration early and therefore *prevent* critical episodes. Continuous ward monitoring seems likely to identify patients who are getting into trouble before they become critical, thus moving us beyond ‘failure to rescue'^121^ to *prevention* of critical events.

Battery-powered untethered vital sign monitoring systems are already available for ward use. Using them will be the first step towards detecting instability in ward patients early enough to intervene effectively.^122^, ^123^, ^124^, ^125^ Continuous ward monitoring detects vital sign abnormalities that are missed with conventional intermittent assessments.^110^ However, it remains unknown whether detecting otherwise missed events prompts effective interventions, much less whether intervening early improves outcomes. Robust trials of continuous ward monitoring are needed.

Vital signs can now be remotely monitored after patients are discharged from the hospital using various wearable systems.^126^^,^^127^ Much research is needed to validate systems for remote automated monitoring and determine whether remote monitoring improves outcomes in patients recovering from surgery at home.

## Strengths and limitations

We used a well-established modified Delphi process combining literature review with expert interpretation. The practical consensus statements and recommendations focus on clinical areas in which optimal diagnostic or therapeutic approaches remain unclear. This methodology does not include a formal systematic literature review or meta-analysis. The process is partly based on expert interpretation and, although the diverse group of experts was carefully selected, remains a discussion between a limited sample of clinicians. There thus remains some risk of bias. Our panel did not include lay members or patient representatives of the target population (i.e. patients who need or had noncardiac surgery). We did not formally document iterations of statements and recommendations during their review and revision in the alternating small-group (arterial pressure management group only) and plenary (whole group) sessions. We used the GRADE framework^10^ but did not formally document the process of agreeing on the classification of the strength of recommendations and the quality of evidence. Although a formal strength of evidence scoring system was not used, the wording of practice recommendations as defined here gives an indication of the group's opinion on the strength of evidence underlying those statements. We highlight areas of uncertainty or persisting discord in the explanatory text accompanying the consensus statements and recommendations. Voting by attendees of the Evidence Based Perioperative Medicine (EBPOM) 2023 World Congress cannot be considered formal expert review, but provided input from an international group of practitioners.

## Conclusions

In adults having noncardiac surgery, intraoperative mean arterial pressures <60–70 mm Hg or systolic arterial pressures <90–100 mm Hg are associated with acute kidney injury, myocardial injury, myocardial infarction, and death. Whether these associations are causal remains largely unknown because there are so far only a few randomised trials on the effect of targeted arterial pressure management on postoperative outcomes, none of which clearly identifies a harm threshold.

We recommend keeping intraoperative mean arterial pressure ≥60 mm Hg in at-risk patients. We further recommend increasing mean arterial pressure targets when venous or compartment pressures are elevated, and treating hypotension based on presumed underlying causes (including vasodilation, hypovolaemia, bradycardia, and low cardiac output). When intraoperative hypertension is treated, we recommend doing so carefully to avoid hypotension. Clinicians should consider continuous intraoperative arterial pressure monitoring as it helps reduce the severity and duration of hypotension compared to intermittent arterial pressure monitoring. Postoperative hypotension is often unrecognised and could be more important than intraoperative hypotension because it is often prolonged. Future research should focus on identifying patient-specific and organ-specific hypotension harm thresholds and optimal treatment strategies for intraoperative hypotension including individualised choice of vasopressors. Research is also needed to guide monitoring and management strategies for recognising, preventing, and treating postoperative hypotension.

## Authors’ contributions

Participation in consensus conference, drafting of manuscript, critical revision of article for important intellectual content, final approval of the version to be published, and agreement to be accountable for all aspects of the work, thereby ensuring that questions related to the accuracy or integrity of any part of the work are appropriately investigated and resolved: all authors.

## Declarations of interest

BS is a consultant for and has received institutional restricted research grants and honoraria for giving lectures from Edwards Lifesciences (Irvine, CA, USA), is a consultant for Philips North America (Cambridge, MA, USA) and has received honoraria for giving lectures from Philips Medizin Systeme Böblingen (Böblingen, Germany), has received institutional restricted research grants and honoraria for giving lectures from Baxter (Deerfield, IL, USA), is a consultant for and has received institutional restricted research grants and honoraria for giving lectures from GE Healthcare (Chicago, IL, USA), has received institutional restricted research grants and honoraria for giving lectures from CNSystems Medizintechnik (Graz, Austria), is a consultant for Maquet Critical Care (Solna, Sweden), has received honoraria for giving lectures from Getinge (Gothenburg, Sweden), is a consultant for and has received institutional restricted research grants and honoraria for giving lectures from Pulsion Medical Systems (Feldkirchen, Germany), is a consultant for and has received institutional restricted research grants and honoraria for giving lectures from Vygon (Aachen, Germany), is a consultant for and has received institutional restricted research grants from Retia Medical (Valhalla, NY, USA), has received honoraria for giving lectures from Masimo (Neuchâtel, Switzerland), is a consultant for Dynocardia (Cambridge, MA, USA), has received institutional restricted research grants from Osypka Medical (Berlin, Germany), and was a consultant for and has received institutional restricted research grants from Tensys Medical (San Diego, CA, USA). BS is an editor of the *British Journal of Anaesthesia*. NF has received honoraria for consulting and lectures from Edwards Lifesciences (Irvine, CA, USA). TJG has received honoraria from Eagle Pharmaceuticals (Woodcliff Lake, NJ, USA), Edwards Lifesciences (Irvine, CA, USA), Medtronic (Minneapolis, MN, USA), and Merck (Rahway, NJ, USA). MPWG has received grant support from the National Institute of Health Research (Leeds, UK), Bill and Melinda Gates Foundation (Seattle, WA, USA), National Lottery Fund (Watford, UK), NHS England (London, UK), Edwards Lifesciences (Newbury, UK), has received consulting fees (Medical Advisory Board and Trial Monitoring) from Edwards Lifesciences (Newbury, UK), Sphere Medical (London, UK), and South West Sensor (Southampton, UK); and is in part funded by the NIHR Senior Investigator Scheme and in part by the NIHR Southampton Biomedical Research Centre. PSM is supported by an Australian National Health and Medical Research Council Investigator grant (ID2008079), Canberra, ACT, Australia. DIS has received research funding from Edwards Lifesciences (Irvine, CA, USA); he also is an advisor and has an equity interest in Perceptive Medical (Newport Beach, CA, USA).

## Funding

The PeriOperative Quality Initiative (POQI) is a 501(c)(3) not-for-profit organisation incorporated in New York, NY, USA. POQI has received funding from both commercial and non-commercial partners, from organisations with an interest in advancing patient safety and quality of care for patients undergoing all types of surgery. This funding comes in the form of unrestricted educational grants. Specifically, there is no understanding that this support is associated with any kind of reciprocal influence, either commercially or in the content and delivery of POQI activities and published material. All POQI papers to date have been published in high-quality peer-reviewed journals. A list of those commercial organisations who have supported POQI to date is as follows: Abbott, Abiomed, Acacia, Astellas, Baxter, CalciMedica, Edwards Lifesciences, Estor, Fresenius, Heron, Inoviva, La Jolla, Mallinckrodt, Masimo, Medasense, Medinspire, Medtronic, Retia, Spectral, Trevena, TopMedTalk, and Toray.
