# Supplementary material for: PeriOperative Quality Initiative (POQI) international consensus statement on perioperative arterial pressure management
Source: Br J Anaesth. 2024 Jun 4;133(2):264–76. doi: 10.1016/j.bja.2024.04.046 (PMC11282474; doi:10.1016/j.bja.2024.04.046)
Supplement: Multimedia component 1 [file mmc1.pdf]

Perioperative Quality Initiative XI (POQI XI) Workgroup Members

Georg Auzinger AFICM FRCP EDIC, Department Chair - Critical Care, Cleveland Clinic London, Professor of Medicine, Cleveland Clinic Lerner College of Medicine, Reader in Critical Care King's College London, 40 Grosvenor Place, London, UK.
No disclosures

Desirée Chappell, CRNA, Vice President Clinical Quality, NorthStar Anesthesia, Irving, TX, USA.
No disclosures

Tong J Gan, MD, MBA, MHS, Helen Shafer Fly Distinguished Professor & Head, Division of Anesthesiology, Critical Care and Pain Medicine, University of Texas MD Anderson Cancer Center, Houston, TX, USA.
Dr Gan has received honoraria from Eagle Pharmaceuticals (Woodcliff Lake, NJ, USA), Edwards Lifesciences (Irvine, CA, USA) Medtronic (Minneapolis, MN, USA), and Merck (Rahway, NJ, USA)

Mark Edwards MB FRCA, Perioperative & Critical Care Research Group, NIHR Southampton Biomedical Research Centre, University Hospital Southampton NHS Foundation Trust / University of Southampton, Southampton, UK.
Dr Edwards has received an honorarium for lecturing for Edwards Lifesciences, is Chief Investigator of the NIHR-funded FLO-ELA trial of cardiac output-guided haemodynamic therapy in patients undergoing emergency gastrointestinal surgery, and is Deputy Chief Investigator of the OPTIMISE II trial of cardiac output-guided haemodynamic therapy in patients undergoing elective gastrointestinal surgery (funded by NIHR and Edwards Lifesciences although does not receive any financial support in this role).

Nick Fletcher MBBS FRCA FFICM, Chair of the Institute of Anaesthesia and Critical Care, Cleveland Clinic London, Professor of Anesthesiology, Cleveland Clinic Lerner College of Medicine, Cleveland Clinic London, UK.

Prof Fletcher is a consultant for Edwards Lifesciences.

Lui G Forni, Intensive Care Unit, Royal Surrey County Hospital NHS Foundation Trust, Department of Clinical & Experimental Medicine, School of Biosciences & Medicine, University of Surrey, UK.

Dr Forni has received research support from Baxter, Ortho Clinical and Sphigotec. Honoraria from Baxter, Fresenius, Sphingotec and Exthera.

Michael P W Grocott MD FRCA FRCP, Perioperative and Critical Care Theme, NIHR Southampton Biomedical Research Centre, University Hospital Southampton / University of Southampton, Southampton, UK.

Dr Grocott has received grant support from the National Institute of Health Research (Leeds, UK), Bill and Melinda Gates Foundation (Seattle, Washington, US), National Lottery Fund (Watford, UK), NHS England (London, UK), Edwards Lifesciences (Newbury, UK), has received consulting fees (Medical Advisory Board and Trial Monitoring) from Edwards Lifesciences (Newbury, UK), Sphere Medical (London, UK), and SouthWestSensor (Southampton, UK); and is in part funded by the NIHR Senior Investigator Scheme and in part by the NIHR Southampton Biomedical Research Centre.

Gudrun Kunst MD PhD EDAIC FRCA FFICM, Consultant Anaesthetist and Professor of Cardiovascular Anaesthesia, Department of Anaesthetics and Pain Therapy, King’s College

Hospital NHS Foundation Trust and School of Cardiovascular and Metabolic Medicine & Sciences, King's College London, UK.
Honoraria and travel expenses from Edwards Lifesciences.

Timothy E. Miller MB ChB FRCA, Department of Anesthesiology, Duke University Medical Center, Durham, NC, USA.
Dr Miller is a consultant for Philips and Retia Medical.

Vicki Morton-Bailey, DNP, AGNP-BC, Director, Clinical and Quality Outcomes, Providence Anesthesiology Associates, Charlotte, NC, USA.
Vicki Morton-Bailey is a speaker for Edwards Lifesciences.

Paul S. Myles, Alfred Hospital and Monash University, Australia.
Dr. Myles is supported by an Australian National Health and Medical Research Council (Canberra, Australia) Investigator Grant.

Marlies Ostermann, King's College London, Guy's & St Thomas Hospital, Department of Critical Care, London, UK.
No disclosures

Jacob Raphael, MD FAHA, Professor of Anesthesiology, Thomas Jefferson University, Sidney Kimmel Medical College, Philadelphia, PA, USA.
Dr. Raphael received consulting fees from Octapharma.

Bernd Saugel, Department of Anesthesiology, Center of Anesthesiology and Intensive Care Medicine, University Medical Center Hamburg-Eppendorf, Hamburg, Germany.

Bernd Saugel is a consultant for and has received institutional restricted research grants and honoraria for giving lectures from Edwards Lifesciences (Irvine, CA, USA). Bernd Saugel is a consultant for Philips North America (Cambridge, MA, USA) and has received honoraria for giving lectures from Philips Medizin Systeme Böblingen (Böblingen, Germany). Bernd Saugel has received institutional restricted research grants and honoraria for giving lectures from Baxter (Deerfield, IL, USA). Bernd Saugel is a consultant for and has received institutional restricted research grants and honoraria for giving lectures from GE Healthcare (Chicago, IL, USA). Bernd Saugel has received institutional restricted research grants and honoraria for giving lectures from CNSystems Medizintechnik (Graz, Austria). Bernd Saugel is a consultant for Maquet Critical Care (Solna, Sweden). Bernd Saugel has received honoraria for giving lectures from Getinge (Gothenburg, Sweden). Bernd Saugel is a consultant for and has received institutional restricted research grants and honoraria for giving lectures from Pulsion Medical Systems (Feldkirchen, Germany). Bernd Saugel is a consultant for and has received institutional restricted research grants and honoraria for giving lectures from Vygon (Aachen, Germany). Bernd Saugel is a consultant for and has received institutional restricted research grants from Retia Medical (Valhalla, NY, USA). Bernd Saugel has received honoraria for giving lectures from Masimo (Neuchâtel, Switzerland). Bernd Saugel is a consultant for Dynocardia (Cambridge, MA, USA). Bernd Saugel has received institutional restricted research grants from Osypka Medical (Berlin, Germany). Bernd Saugel was a consultant for and has received institutional restricted research grants from Tensys Medical (San Diego, CA, USA). Bernd Saugel is an Editor of the British Journal of Anaesthesia.

Daniel I. Sessler MD, Michael Cudahy Professor and Chair, Department of Outcomes Research, Cleveland Clinic, Cleveland, OH, USA.

Dr Sessler has received research funding from Edwards Lifesciences; he also is an advisor and has equity interest in Perceptive Medical (Newport Beach, CA, USA).

Andrew D Shaw MB FRCA FFICM FRCPC, Professor and Chair, Department of Intensive Care and Resuscitation, Cleveland Clinic, Cleveland, OH, USA.

Dr Shaw has served as a consultant for Novartis, Alexion, AM Pharma, Renibus, Retia.

Alexander Zarbock, Department of Anesthesiology, Intensive Care and Pain Medicine, University Hospital Münster, Münster, Germany.

Dr Zarbock is a consultant for Bayer, Novartis, Renibus, Guard Therapeutics, AM Pharma, BioMerieux, Baxter, and Paion. He received lecture fees from BioMerieux, Baxter, and Paion.
